# Guideline‐directed medical therapy assessment in heart failure patients undergoing percutaneous mitral valve repair

**DOI:** 10.1002/ehf2.14705

**Published:** 2024-02-13

**Authors:** Karl‐Patrik Kresoja, Marianna Adamo, Karl‐Phillipp Rommel, Lukas Stolz, Nicole Karam, Cristina Giannini, Bruno Melica, Ralph Stephan von Bardeleben, Christian Butter, Patrick Horn, Fabien Praz, Daniel Kalbacher, Christos Iliadis, Holger Thiele, Jörg Hausleiter, Marco Metra, Philipp Lurz

**Affiliations:** ^1^ Department of Cardiology University Medical Center of Mainz Mainz Germany; ^2^ Department of Internal Medicine/Cardiology Heart Center Leipzig at University of Leipzig and Leipzig Heart Science Strümpellstraße 39 Leipzig 04289 Germany; ^3^ Cardiac Catheterization Laboratory and Cardiology, ASST Spedali Civili and Department of Medical and Surgical Specialties, Radiological Sciences and Public Health University of Brescia Brescia Italy; ^4^ Medizinische Klinik und Poliklinik I Klinikum der Universität München Munich Germany; ^5^ Department of Cardiology European Hospital Georges Pompidou and Paris Cardiovascular Research Center, INSERM U970 Paris France; ^6^ Cardiothoracic and Vascular Department Azienda Ospedaliero‐Universitaria Pisana Pisa Italy; ^7^ Centro Hospitalar Vila Nova de Gaia Espinho Portugal; ^8^ Department of Cardiology, Immanuel Heart Center Bernau Brandenburg Medical School Theodor Fontane Bernau Germany; ^9^ Department of Cardiology, Heart Center University Hospital of Düsseldorf Düsseldorf Germany; ^10^ Universitätsklinik für Kardiologie, Inselspital Bern Bern Switzerland; ^11^ Department of Cardiology University Heart and Vascular Center Hamburg Hamburg Germany; ^12^ German Center for Cardiovascular Research (DZHK), partner site Hamburg/Kiel/Lübeck Hamburg Germany; ^13^ Department of Cardiology, Heart Center University Hospital of Cologne Cologne Germany

**Keywords:** Mitral regurgitation, Guideline‐directed medical therapy, Heart failure with reduced ejection fraction

## Abstract

**Aims:**

Achieving optimized guideline‐directed medical therapy (GDMT) is recommended prior to transcatheter mitral valve edge‐to‐edge repair (M‐TEER) for secondary mitral regurgitation (SMR). We aimed to propose and validate an easy‐to‐use score for assessing the quality of GDMT in patients with heart failure with reduced ejection fraction (HFrEF) undergoing M‐TEER.

**Methods and results:**

Among the 1641 EuroSMR patients enrolled in the EuroSMR Registry who underwent M‐TEER, a total of 1072 patients [median age 74, interquartile range (IQR) 67–79 years, 29% female] had complete data on GDMT and a left ventricular ejection fraction ≤ 40% and were included in the current study. We proposed a GDMT score that considers the dosage levels of three medication classes (angiotensin‐converting enzyme inhibitors/angiotensin receptor blockers/angiotensin receptor‐neprilysin inhibitors, beta‐blockers, and mineralocorticoid receptor antagonists), with a maximum score of 12 points indicating optimal GDMT. The primary outcome was all‐cause mortality. The median GDMT score was 4 points (IQR 3–6). All three domains of the scoring system were associated with all‐cause mortality (*P* < 0.05 for all). The overall GDMT score was associated with all‐cause mortality (hazard ratio 0.90, 95% confidence interval 0.86–0.95 for each 1‐point increase in the GDMT score). This association remained significant after adjusting for renal function and co‐morbidities.

**Conclusions:**

This study demonstrates the utility of a simple GDMT scoring system for assessing the adequacy of GDMT in HFrEF patients with relevant SMR undergoing M‐TEER. The GDMT score has potential applications in guiding the design of future clinical trials and aiding clinical decision‐making processes.

## Background and aims

Secondary mitral regurgitation (SMR) is frequent among patients with heart failure with reduced ejection fraction (HFrEF) and is associated with a dismal prognosis.^1^, ^2^ In current guidelines, transcatheter mitral valve edge‐to‐edge repair (M‐TEER) has been recommended as a therapeutic option for eligible patients.^3^, ^4^, ^5^ These guidelines emphasize the importance of optimization of guideline‐directed medical therapy (GDMT) for HFrEF before referring patients to M‐TEER.^5^, ^6^ However, real‐word data often show the underuse of GDMT.^1^, ^7^ Recent evidence highlighted that optimal GDMT is only achieved when maximum trial doses of substances are prescribed, which is often not the case in clinical practice. Thus, both initiation and titration to target doses administered in randomized controlled clinical trials are related to a better outcome for patients with heart failure.^7^, ^8^, ^9^, ^10^ Yet, given the large number of substances that are recommended and available for GDMT, it is challenging to provide an objective measure of GDMT that allows for monitoring of therapy intensity and to allow for comparison in scientific studies.

So far, there is no uniform framework on how to grade the quality of GDMT for HFrEF patients. We therefore aimed to provide a simple scoring framework to assess the quality of GDMT in HFrEF patients in general, validate it in a cohort of HFrEF patients with SMR undergoing M‐TEER, and assess its association with all‐cause mortality.

## Methods

### Study population

This analysis included patients with SMR who underwent M‐TEER at 11 European centres from the EuroSMR Registry. The EuroSMR design has been published before.^11^, ^12^ The management of GDMT was left to local physicians. All patients gave their informed consent. The study complied with the Declaration of Helsinki and was performed with the approval of local ethical committees. Patients with a left ventricular ejection fraction (LVEF) > 40% and patients in whom GDMT information was incomplete at baseline were excluded.

### Guideline‐directed medical therapy score

**Figure 1:**
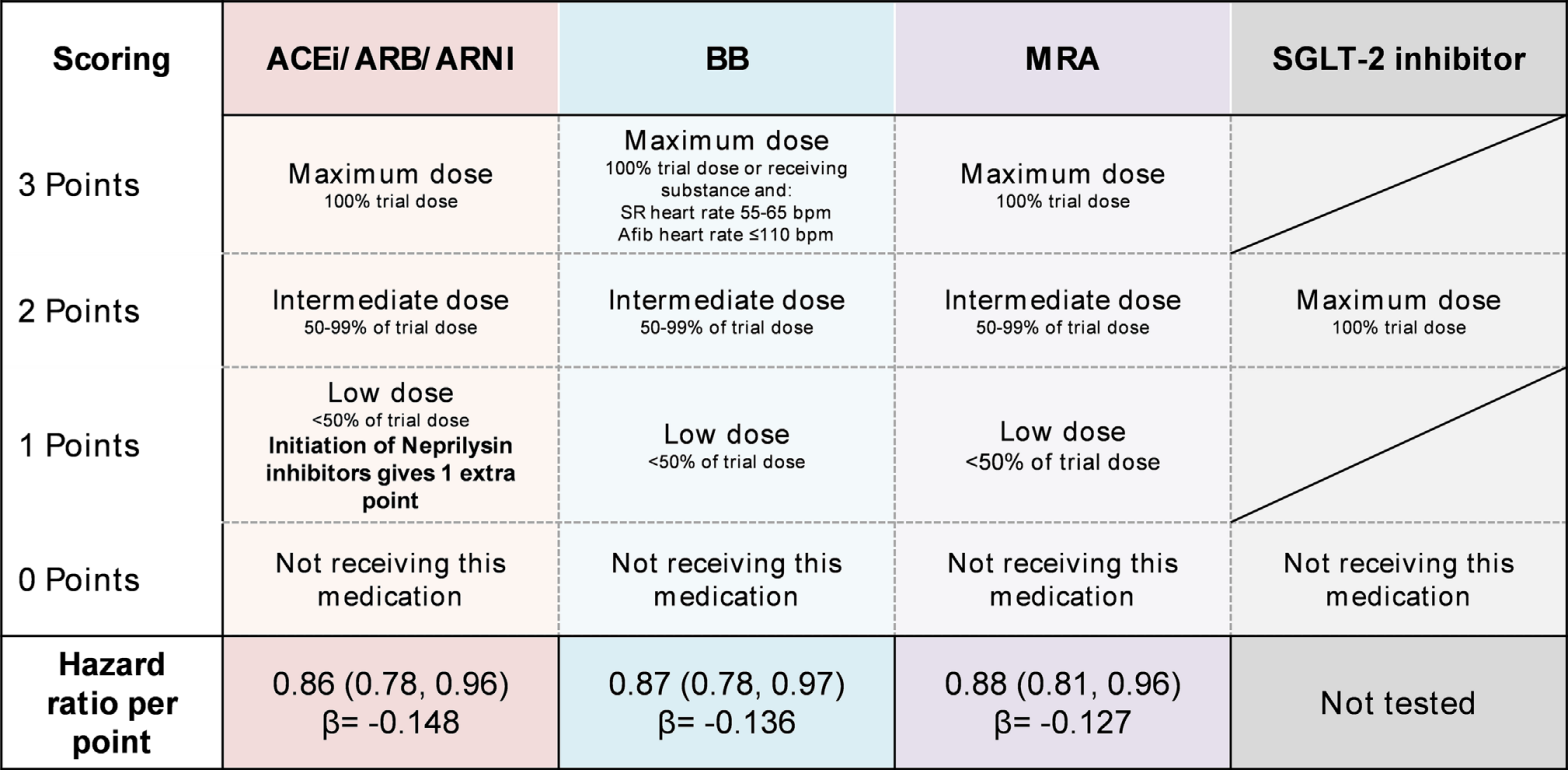
Framework for a scoring system for guideline‐directed medical therapy (GDMT). Proposed scoring scheme for a score to quantitatively assess the quality and quantity of GDMT. Patients can receive points from each domain and can receive a maximum of up to 12 points in total, indicating maximum GDMT. Further, the association of the domains with all‐cause mortality is displayed. Of note, sodium‐glucose cotransporter‐2 (SGLT‐2) inhibitors were not validated for the proposed score. Trial doses were defined according to the guidelines of the European Society of Cardiology Guidelines for heart failure.^6^ ACE‐I, angiotensin‐converting enzyme inhibitor; Afib, atrial fibrillation; ARB, angiotensin receptor blocker; ARNI, angiotensin receptor‐neprilysin inhibitor; BB, beta‐blocker; MRA, mineralocorticoid receptor antagonist; SR, sinus rhythm.

As shown in *Figure* *1*, GDMT medications were stratified into four classes: (i) angiotensin‐converting enzyme inhibitors (ACE‐Is), angiotensin receptor blockers (ARBs), or angiotensin receptor‐neprilysin inhibitors (ARNIs); (ii) beta‐blockers (BBs); (iii) mineralocorticoid receptor antagonists (MRAs); and (iv) sodium‐glucose cotransporter‐2 (SGLT‐2) inhibitors. A scoring system was proposed where patients could receive 0–3 points for each category according to the dosage levels [0 for not receiving the substance, 1 for minimum dosage (<50% of recommended trial dose), 2 for intermediate dosage (50–99% of recommended trial dose), and 3 for maximum dosage (100% of recommended trial dose); 1 extra point was given for ARNI initiation, and there were exceptions for BBs and MRAs] (*Figure* *1*). Of note, SGLT‐2 inhibitors were not tested in the present study due to a lack of availability in the study period.

### Statistical analysis and matching

Data are provided as the median and corresponding interquartile range (IQR) or the mean with standard deviation. Continuous variables were compared with the Mann–Whitney *U* test or the Kruskal–Wallis test, where appropriate. Categorical variables were compared using Fisher's exact test.

Cox regression analyses were performed to test the prognostic relevance of the baseline GDMT score with regard to all‐cause mortality.

A two‐sided significance level of α 0.05 was defined as appropriate to indicate statistical significance. Statistical analyses were performed using the SPSS software (IBM Corp. released 2017, Version 25.0, Armonk, NY, USA).

## Results

Of 1641 patients undergoing M‐TEER between 2008 and 2021, 482 were excluded due to an LVEF > 40% and 87 due to missing data on the quality or quantity of GDMT. This resulted in a final cohort of 1072 patients, which exhibited characteristics similar to previous M‐TEER cohorts, including increased perioperative risk, impaired renal function, and significant SMR and left ventricular dilatation (*Table* *1*).

**Table 1:** Baseline characteristics of the cohort according to a guideline‐directed medical therapy score below or above the median of the points

	Overall cohort	GDMT score ≤ 4	GDMT score > 4	*P*‐value
	*N* = 1072	*N* = 614	*N* = 458	
Age, years	74 (67–79)	75 (69–80)	73 (64–78)	<0.001
Female sex, %	307 (29)	181 (30)	126 (28)	0.50
NYHA class III/IV, %	939 (87)	536 (87)	403 (87)	0.67
BMI, kg/m^2^	26 (23–30)/1067	26 (23–29)/611	27 (24–32)/456	<0.001
EuroSCORE II, %	9.6 (5.0–23.0)/376	9.2 (5.0–22)/235	11 (5–24)/141	0.76
Estimated glomerular filtration rate, mL/min/1.73 m^2^	47 (33–64)/1001	45 (30–60)/565	49 (36–67)/436	<0.001
NT‐proBNP, pg/mL	3344 (1658–7291)/660	3500 (1786–7420)/379	3100 (1547–6698)/281	0.11
Mean arterial blood pressure, mmHg	87 (77–97)/1003	87 (77–100)/606	85 (77–95)/397	0.004
Ischaemic heart disease, %	576 (57)/1009	349 (60)/586	227 (54)/423	0.071
Diabetes, %	363 (35)/1025	191 (32)/595	172 (40)/430	0.010
Arterial hypertension, %	702 (70)/1006	392 (67)/584	310 (74)/422	0.031
Previous myocardial infarction, %	355 (33)/1063	210 (35)/609	145 (32)/454	0.39
Previous percutaneous coronary intervention, %	391 (47)/839	250 (49)/515	141 (44)/324	0.18
Previous coronary artery bypass grafting, %	224 (22)/1017	124 (21)/592	100 (24)/425	0.36
Previous stroke, %	104 (10)	60 (10)	44 (10)/457	1.00
Chronic obstructive pulmonary disease, %	175 (16)/1067	109 (18)/612	66 (15)/455	0.16
Atrial fibrillation, %	630 (59)/1068	363 (60)/610	267 (58)	0.71
Mitral regurgitation severity				0.068
II	64 (5)	44 (7)	20 (4)	
III	1008 (95)	570 (93)	438 (96)	
Mitral valve effective regurgitant orifice area, cm^2^	0.29 (0.2–0.39)/812	0.30 (0.20–0.40)/456	0.27 (0.20–0.38)/356	0.077
Mitral valve biplane vena contracta, mm	6.9 (5.5–8.0)/647	7 (5.7–8.1)/356	6.5 (5.4–7.6)/291	0.001
Concomitant severe TR, %	190 (18)/1039	126 (21)/591	64 (14)/448	0.004
Left ventricular end‐diastolic volume, mL	193 (150–240)/984	148 (188–234)/549	203 (152–253)/435	0.004
Left ventricular ejection fraction, %	30 (24–35)	30 (24–35)	29 (23–34)	0.030
Tricuspid annulus plane systolic excursion, mm	16 (14–19)/850	16 (13–19)/475	17 (14–19)/375	0.78
Estimated systolic pulmonary artery pressure, mmHg	48 (38–58)/900	47 (38–58)/520	48 (39–58)/380	0.48

BMI, body mass index; GDMT, guideline‐directed medical therapy; NT‐proBNP, N‐terminal pro‐brain natriuretic peptide; NYHA, New York Heart Association; TR, tricuspid regurgitation.

In case of missing data, the number of available observations is given as /*n*, whereas *n* is the number of available observations.

**Figure 2:**
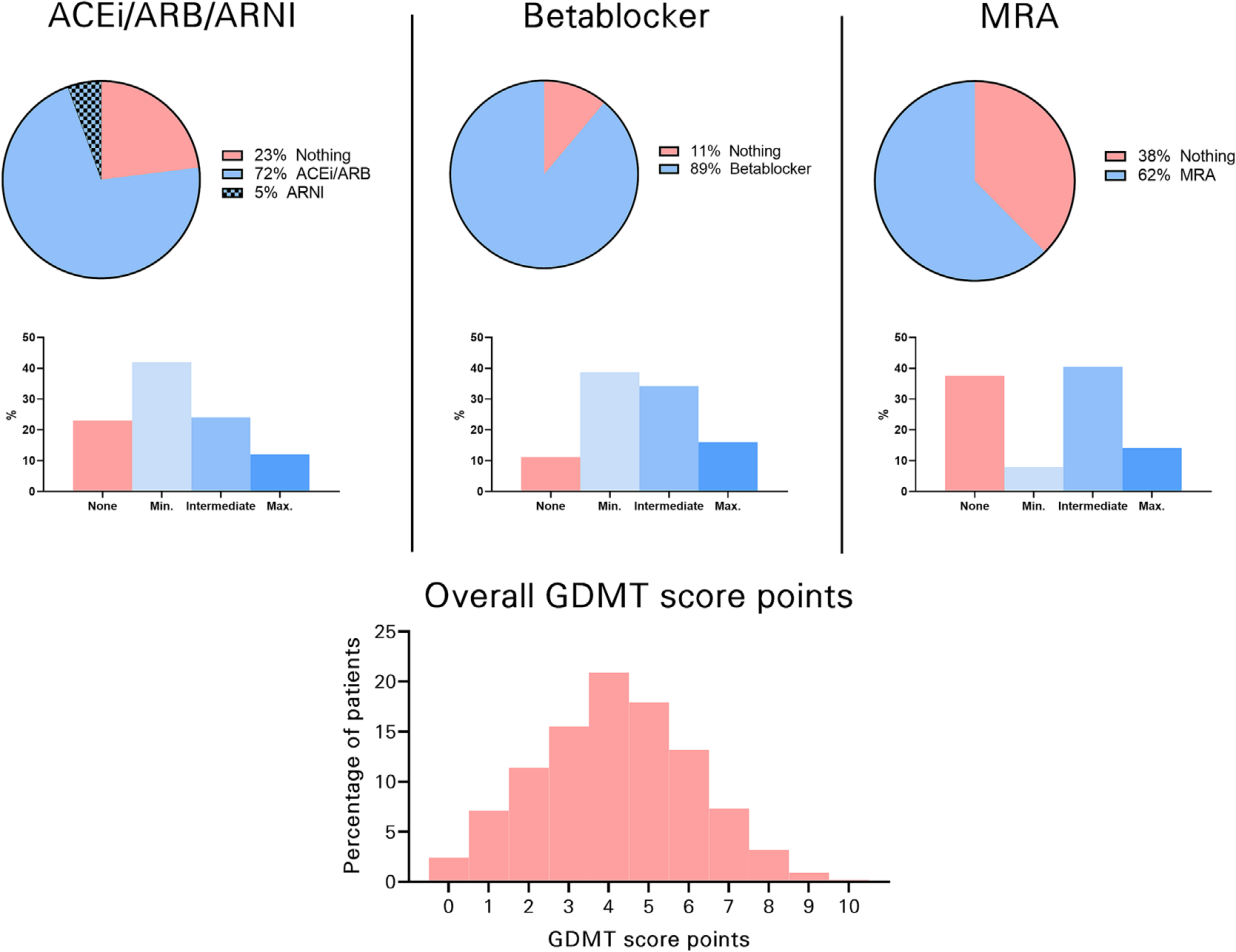
Quality and quantity of guideline‐directed medical therapy (GDMT) in the EuroSMR cohort. The pie charts represent the fraction of patients receiving the medication at all, and the bar charts below show the corresponding intensity of medication with reference to the recommended study trial dose. ACE‐I, angiotensin‐converting enzyme inhibitor; ARB, angiotensin receptor blocker; ARNI, angiotensin receptor‐neprilysin inhibitor; MRA, mineralocorticoid receptor antagonist.

The GDMT score was calculated as illustrated in *Figure* *1*, and the distribution and intensity of GDMT according to the scoring system are displayed in *Figure* *2*. The median GDMT score was 4 (IQR 3–5) points, indicating low GDMT intensity before M‐TEER. Only 12 patients (1.1%) achieved a GDMT score of 9 points, which is required to reach trial GDMT dosages in all three domains.

During a median follow‐up of 604 (IQR 342–1105) days, the primary outcome of all‐cause mortality occurred in 412 patients (38.4%). All score categories were associated with the primary outcome (*Figure* *1*; *P* < 0.05 for all). The standardized beta values between the three score categories were comparable, indicating a reasonable fit of the point scoring system (ACE‐I/ARB/ARNI, β = −0.15; BB, β = −0.14; and MRA, β = −0.13). The overall GDMT score was also associated with the primary outcome [hazard ratio (HR) 0.90, 95% confidence interval (CI) 0.86–0.95 for each increase in one GDMT score point]. The GDMT score remained associated with the primary outcome after adjustment for estimated glomerular filtration rate (HR_adj_ 0.92, 95% CI 0.87–0.96) and the EuroSCORE II (HR_adj_ 0.91, 95% CI 0.83–0.99).

## Conclusions

This study introduces a simple scoring system to assess the quality of GDMT in HFrEF patients in general and was validated in an HFrEF population with SMR undergoing M‐TEER. The purpose of this scoring system is not to predict prognosis but rather to provide a framework to grade the quality of GDMT, to allow for easy monitoring of GDMT adherence, on the one hand, and for comparability in the setting of clinical trials, on the other hand.

While the rate of administration of the different GDMT classes was comparable with randomized trials,^4^ GDMT intensity was low in this patient population, with almost no patients receiving maximum GDMT. With recent evidence showing that GDMT dosing and up‐titration is a crucial prognostic factor for HFrEF patients,^9^ the need for a tool to assess GDMT becomes evident.^8^ Interestingly, the self‐perception of physicians is in dire contrast to clinical reality, where most cardiologists believe their patients to be on optimal GDMT.^13^ The reasons for non‐up‐titration are manifold, but one important aspect might be the lack of perception of non‐optimized GDMT; a scoring system that automatically provides feedback on non‐optimal GDMT adherence might help to further sharpen physicians' attention in the clinical setting and raise awareness about GDMT intensification.^8^

Among objective criteria, impaired renal function and hypotension are often cited as common reasons for suboptimal dosing of GDMT. In line with this, renal function was a significant predictor of outcomes in patients treated conservatively in the COAPT study but not in those undergoing M‐TEER,^14^ suggesting that at least parts of the adverse effects of renal impairment can be alleviated by M‐TEER. Even in the absence of maximum GDMT, M‐TEER might provide clinical benefits by improving haemodynamics, especially with regard to forward flow and systemic perfusion, allowing for further intensification of GDMT, which has been associated with lower mortality.^11^, ^15^ Importantly, for some patients, M‐TEER might allow subsequent GDMT up‐titration. A scoring system in this setting might also be important for future randomized trials to allow for objective comparison and changes in GDMT adherence consequently to interventions that affect haemodynamics like M‐TEER.

The study's strengths include its large sample size, multicentre design, and use of real‐world data. However, the study only included patients undergoing M‐TEER, which may limit generalizability to other patient populations. Additionally, the availability of certain medications, such as ARNIs and SGLT‐2 inhibitors, varied during the study period and could therefore not be validated in the GDMT score. Yet, given the ease and safety of SGLT‐2 inhibitor initiation, directly at the GDMT recommended dose and without relevant adverse effects on renal function and haemodynamics, we believe this to be a justifiable limitation of the study.^16^ Lastly, reasons for suboptimal up‐titration of GDMT were not available and could not reasonably be assessed given the retrospective nature of the study.

In conclusion, this study provides a way to score GDMT intensity in patients with HFrEF and SMR undergoing M‐TEER. This scoring framework might be used in future clinical trials by heart teams to assess the quality of GDMT in this patient population. Lastly, the study highlights the need for further research to improve the use of GDMT in these patients.

## Conflict of interest

K.‐P.K. has been a consultant to Edwards Lifesciences. M.A. has been a consultant to Abbott Structural Heart and Medtronic. K.‐P.R. reports no conflict of interest. L.S. has received speaker honoraria from Edwards Lifesciences. N.K. has received consultant fees from Abbott Structural Heart, Edwards Lifesciences, and Medtronic. C.G. has received consultant honoraria from Medtronic. B.M. has received a consulting fee and honoraria for lectures from Abbott and from Edwards. R.S.v.B. is a consultant to Abbott, Edwards, Jenscare, Medtronic, NeoChord, Philips, and Siemens. C.B. reports no conflict of interest. P.H. has received travel support and an educational grant from Abbott Medical GmbH and Edwards Lifesciences and an unrestricted research grant from Edwards Lifesciences. F.P. has received travel expenses from Abbott Vascular, Polares Medical, and Edwards Lifesciences. D.K. has received personal fees from Abbott Medical, Edwards Lifesciences, and Pi‐Cardia Ltd. C.I. has received travel and consultant honoraria from Abbott and Edwards Lifesciences. H.T. reports no conflict of interest. J.H. has been a consultant to Edwards Lifesciences. M.M. has received the following personal fees of minimal amounts since January 2021: from Amgen, LivaNova, and Vifor pharma as a member of Executive or Data Monitoring Committees of sponsored clinical trials; and from AstraZeneca, Bayer, Boehringer Ingelheim, Edwards Lifesciences, and Roche Diagnostics for participation to advisory boards and/or speeches at sponsored meetings. P.L. has been a consultant to Abbott Structural Heart, Edwards Lifesciences, and Medtronic.

## Funding

None.
